# Supplementary material for: Qualitative cross-country comparison of whether, when and how people diagnosed with lung cancer talk about cigarette smoking in narrative interviews
Source: BMJ Open. 2018 Nov 12;8(11):e023934. doi: 10.1136/bmjopen-2018-023934 (PMC6252649; doi:10.1136/bmjopen-2018-023934)
Supplement: Author's manuscript [file bmjopen-2018-023934.draft_revisions.pdf]

BMJ Open is committed to open peer review. As part of this commitment we make the peer review history of every article we publish publicly available.

When an article is published we post the peer reviewers' comments and the authors' responses online. We also post the versions of the paper that were used during peer review. These are the versions that the peer review comments apply to.

The versions of the paper that follow are the versions that were submitted during the peer review process. They are not the versions of record or the final published versions. They should not be cited or distributed as the published version of this manuscript.

BMJ Open is an open access journal and the full, final, typeset and author-corrected version of record of the manuscript is available on our site with no access controls, subscription charges or pay-per-view fees (http://bmjopen.bmj.com).

If you have any questions on BMJ Open's open peer review process please email info.bmjopen@bmj.com

BMJ Open

The Elephant in the room? A cross-country comparison of talk about cigarette smoking among people diagnosed with lung cancer

Journal:	*BMJ Open*
Manuscript ID	bmjopen-2018-023934
Article Type:	Research
Date Submitted by the Author:	02-May-2018

Complete List of Authors:	Hajdarevic, Senada; Umeå University, Department of Nursing<br>Rasmussen, Birgit; The Institute for Palliative Care; Department of Health Sciences, Lund University<br>Overgaard Hasle, Trine; Research Centre for Cancer Diagnosis in Primary Care, Research Unit of General Practice, Aarhus University, Denmark<br>Ziebland, Sue; Health Experiences Research Group, Nuffield Department of Primary Care Health Sciences, University of Oxford

Keywords:	patients' experiences, lung cancer, smoking, health care, qualitative comparative study

SCHOLARONE™
Manuscripts

For peer review only

The Elephant in the room? A cross-country comparison of talk about cigarette smoking among people diagnosed with lung cancer

*Hajdarevic Senada[1], Senior Lecturer

Rasmussen Birgit H[2,3], Professor

Overgaard Hasle Trine L[4], Research assistant

Ziebland Sue[5], Professor

[1]Department of Nursing, Umeå University, Umeå Sweden

[2] The Institute for Palliative Care, Lund University and Region Skåne, Sweden and Lund University, Skane University Hospital, Lund, Sweden

[3]Department of Clinical Sciences Lund, Surgery, Lund, Sweden

[4]Research Centre for Cancer Diagnosis in Primary Care, Research Unit of General Practice, Aarhus University, Aarhus Denmark

[5]Health Experiences Research Group, Nuffield Department of Primary Care Health Sciences, University of Oxford, UK.

Corresponding author:

Senada Hajdarevic,

Department of Nursing, Umeå University,

90187 Umeå, Sweden

senada.hajdarevic@umu.se; phone: +46 (0) 90 786 91 24

fax : + 46 (0) 90-13 53 24

Keywords: health care, lung cancer, patients' experiences, qualitative study, smoking.

Word count, excluding title page, abstract, references, figures and tables: 2868 words

For peer review only

Abstract

Objectives: To compare and examine how patients with lung cancer (LC) in three countries, with different survival rates, raise and talk about cigarette smoking and its relationship with help seeking.

Design: A qualitative cross-country comparison with analysis of in-depth interviews.

Setting: Participants in Sweden, Denmark and England were interviewed during 2015-2016. Interviews, using a narrative approach, were conducted in participants' home by trained and experienced qualitative researchers.

Participants: Seventy two men and women diagnosed with lung cancer were interviewed within six months of their diagnosis.

Results: The English participants, regardless of their own smoking status, typically raised the topic of smoking early in their interviews. Smoking was mentioned in relation to symptom appraisal and interactions with others, including health professionals. Participants in all three countries interpreted their symptoms in relation to their smoking status, but in Sweden (unlike England) there was no suggestion that this deterred them from seeking care. English participants, but not Swedish or Danish, recounted reluctance to consult healthcare professionals with their symptoms while they were still smoking, some gave up shortly before consulting. Some English patients described defensive strategies to challenge stigma or pre-empt other people's assumptions about their culpability for the disease. A quarter of the Danish and 40% of the Swedish participants did not raise the topic of smoking at any point in their interview.

For peer review only

Conclusion: The causal relationship between smoking and LC is well-known in all three countries, yet the links between a sense of responsibility, stigma and reluctance to consult is not inevitable. These findings help illuminate why English patients with LC tend to be diagnosed at a later stage than their Swedish counterparts.

Article summary

Strengths and limitations of this study

- The cross- country qualitative comparative study of three Northern European countries explored how people with lung cancer position their talk about cigarette smoking and the consequences of the association with smoking for their help-seeking
- The topic of smoking was always raised by the participant, not the research team, enabling us to compare when and how they framed their talk about LC and smoking

What is already known on this subject:

- Denmark, England and Sweden have very similar population cigarette smoking rates (18%, 20%, 20% respectively). In year 2005-2009 Sweden had higher one-year survival (40.8%) for lung cancer (LC) than Denmark (35.3%) and England (31.3%).
- Awareness of the relationship between smoking and lung cancer is high in all three countries.
- English and Danish smokers with lung cancer symptoms consult their GP later than non-smokers with the same symptoms.
- Patients with LC in England and Scotland have described an initial reluctance to visit the GP with symptoms, and feelings of shame and blame, which have been linked to the image of LC as a smokers' disease.

For peer review only

What this study adds:

- In the English narratives, accounts of lung cancer are infused with references to smoking. This contrasts particularly with the Swedish interviews, 40% of which did not mention smoking at all.
- By demonstrating differences between patients' narratives of LC in England, Sweden and Denmark the study challenges the notion that there is any inevitability about the link between responsibility, stigma and reluctance to seek care.

Why it is important:

- The causal relationship between smoking and lung cancer is undeniable, yet the consequences of this awareness for patients seeking care is not uniform.
- The study sheds light on findings from international comparisons of cancer survival by offering a new, potentially modifiable, explanation for why LC patients in England may be diagnosed at a later stage

INTRODUCTION

Health problems which are known to be associated with certain elective 'health behaviours' can lead to stigma and victim-blaming [1]. A prominent example is lung cancer (LC): patients in England and Scotland have reported that, on hearing the diagnosis, other people assume that they are (or have been) heavy smokers [2-5]. Images of blackened lungs and television adverts including the personal testimonies of LC patients voicing their regrets for smoking add to the impression of personal culpability [3]. A highly cited qualitative study published in

For peer review only

the BMJ drew attention to the 'stigma, shame and blame' that many people with lung cancer experience due to the disease's association with smoking [3]. This matters for several reasons, including because patients who fear being blamed are deterred from seeking help when they have symptoms. Yet, as we will show, there is no inevitability about the link between perceptions of responsibility, experience of stigma and a reluctance to consult. We draw on findings from our comparative qualitative study of experiences of LC in Sweden, Denmark and England.

Lung cancer is the largest cancer related cause of death worldwide [6]. Denmark, UK and Sweden have similar population smoking rates (~18%, 20%, 20% respectively) [7]. International Cancer Benchmarking data have demonstrated that patients with lung cancer in Denmark and England are diagnosed at a more advanced stage than those in Sweden [8]. Sweden also continues to show a higher one-year survival for LC than Denmark and England [9].

Possible explanations for why people in England and Denmark consult with later stage disease have been considered, including differences in how people interpret bodily sensations, access health care, describe their problem to their doctors and how the doctors respond [9-11]. Sociocultural aspects and characteristics of the health care system influence how and when people seek care [12]. Past experiences of contact with the health care services contribute to expectations and subsequent decisions to seek help [13-18]. Awareness of LC as a smoking related disease is high in all three countries. Smoking in restaurants, bars and workplaces has been banned in Sweden since 2005, Denmark since 2007 and England since 2007, with the legislation reinforcing the reduced social acceptance of smoking.

For peer review only

METHOD

Participants and recruitment

We conducted a cross country comparative qualitative study of interviews collected within six months of a diagnosis of lung cancer (LC) in Denmark (n=22), England (n=20) and Sweden (n=30) [19]. Interviews were conducted during 2015-2016. Purposive sampling [20] through hospital clinics (in Sweden) and also primary care, support organisations, via snowball-sampling and social media (in England and Denmark) sought comparable variation across gender, age, urban and rural locations, and pathway to diagnosis.

Data collection

Participants gave informed consent and decided the place for their interview, usually their own home. The experienced qualitative researchers had backgrounds in nursing (Sweden), sociology (England) and anthropology (Denmark). A semi structured interview guide based on social science theories, and cancer research literature [3, 15, 21-25], was developed to enhance cross country comparisons [26]. All interviews began with an open-ended question: "*Could you start by telling me, in your own words and in as much detail as you want, about what has happened since you first started to suspect there might be a problem with your health?"* followed by flexibly used prompts from an interview guide. We deliberately avoided specific questions about whether the person smoked, or had ever smoked, cigarettes.

Analysis

A specialist computer software (NVivo 10) was used to code, sort and retrieve data. The interview transcripts were read several times before coding. As a first step we noted *if* and

For peer review only - http://bmjopen.bmj.com/site/about/guidelines.xhtml

how early in the interview the topic of smoking was first raised by the participant. Second we used thematic analysis [27] to consider *how* the participants talked about smoking during their interviews. Three authors (SH, SZ and BHR) used a data-driven coding framework developed through discussion at face to face workshop meetings and via phone conferences. We discussed and refined our interpretations of observed similarities and differences between the countries. For this paper direct quotes have been translated into English (and checked by BHR, the tri-lingual member of our team).

Patient involvement

Public and Patient Involvement (PPI) was conducted in accordance with good practice in each country. LC patients in England were involved in the whole process from the preparation of funding application through the development of the study and discussion of preliminary results. PPI members in all three countries commented on the participant information sheets and the interview guide and advised on recruitment strategies.

RESULTS

Differences between countries in the frequency, positioning and content of talk about smoking were immediately apparent. We present our comparative findings first in relation to the point in the interview at which participants raised the topic of smoking, followed by a thematic analysis of the talk about smoking in relation to symptom appraisal, help seeking and communication with others.

For peer review only

If and when smoking was raised by the participant

All but two of the English participants raised the topic of smoking, unprompted, in their interviews; this usually occurred within the opening sections or first 15% of the interview transcript. More than one quarter of the Danish participants and 40% of the Swedish participants did not raise the topic of smoking at all in their interviews; those who did were less likely than their English counterparts to raise it in the early part of their narrative. (please see the Table 1.)

Table1. Participant characteristics and the proportion of interviews where the participant raised the topic of smoking

	Denmark	England	Sweden
Total number of interviewed participants	22	20	30
Women	8 (36%)	10 (50%)	15 (50%)
Age range (*year*) 31-50	0	2	2
51-70	15	12	21
71-90	7	6	7
Participants who raised the topic of smoking within the first 15% of the interview	9 (41%)	11(55%)	9 (30%)
Participants who raised the topic of smoking at any point in their interview	16 (73%)	18 (90%)	18 (60%)

How participants talked about smoking

Participants raised the topic of smoking when talking about how they had interpreted their symptoms and when describing interactions with others, including how their health care professionals responded.

Smoking as a frame to interpret symptoms

For peer review only - http://bmjopen.bmj.com/site/about/guidelines.xhtml

Our first observation is that participants often raised the topic of smoking as if answering an (unasked) question, for example: *"But I mean, if I hadn't of broken me back they wouldn't have found (the cancer). And [er…pause]. The question is, 'Did you stop smoking?' well, Yeah."* (Ex- smoker, ENG L18)

When describing decisions to consult, the never smokers and longer term ex-smokers mentioned their (non)smoking as part of their explanation for not expecting this diagnosis. *"I thought of course that it was pneumonia or something, so I never thought...as we all know, I have never smoked, I have never worked in smoky surroundings. I could never imagine that it could be something like that."* (Never-smoker, SWE LC147)

Long-term ex-smokers often commented they had not been expecting LC because they had stopped smoking many years ago. *"I mean, it never dawned on me that I was - lung cancer, didn't really figure. I have smoked when I was younger. That was 34 years ago."* (Ex-smoker, ENG L12)

People who were still smokers, or had recently stopped, said they had interpreted and normalised their symptoms - a long-term cough was seen as a 'smoker's cough' and a degree of breathlessness as normal for a smoker.

'I had this persistent cough which I recognised as a smoker's cough, like [coughs] and it was constant'. (Ex-smoker, ENG L05)

"You know that you smoke so it's normal with breathlessness when one was walking and exerted oneself a bit too much. So that was something you were expecting since you were smoking. So, therefore, you did not react in some specific way to it." (Current smoker, SWE 105 LC)

For peer review only

"And as long as I was smoking I was also coughing. But it was…it did not get worse. And it also disappeared when I quitted smoking, so I really did not have any indication." (Ex-smoker, SWE 150 LC)

Smokers also justified a reluctance to consult on the grounds that the sensations and symptoms they experienced were not sufficiently serious, that they felt too well, or the signs were not those they associated with LC.

"I always thought lung cancer was due to smoking. [um] And I knew people who had lung cancer and they used to say to me, you know, [um] coughing up a lot of blood, things like that. I went, "Really." And I didn't have none of that. You see. So I never put it down to cancer." (Current smoker, ENG L09)

"I know that I'm smoking and I've been smoking for many years, but I've always had good health and I've never coughed like many do in the morning for many years ... or ... I have never done that. So, I did not really think it could be cancer, because I had it great, and in principle I still have" (Current smoker, DK LC14)

Smoking as a frame when interacting with others

Accounts from some of the English smokers suggested that if health care professionals 'blamed' smoking for the symptoms, this would deter them from taking action. *"... I said to the GP that my Dad and my Uncle had LC and I thought they should have been a bit quicker to check it out. ... Basically it [the symptoms] was worse at night time and everything. First of all I think she [the GP] blamed it on my smoking."* (Current smoker, ENG L03)

We also heard several accounts, particularly from Swedish participants, of health care professionals who had worked hard to avoid any sense of blame in relation to smoking. In the

For peer review only - http://bmjopen.bmj.com/site/about/guidelines.xhtml

first of these examples a Swedish ex-smoker who voiced the concern that smoking might have been responsible was contradicted, kindly, though not entirely convincingly, by the doctor. *"Now we should not mix in smoking in this', the doctor said. And they were saying that in the order that I should not have such a guilty conscience, I can imagine. But she said that there were many non-smokers who get lung cancer,... but obviously they[doctors] think it[smoking] might have contributed, absolutely."* (Recent ex-smoker, SWE 123LC)

"The doctor said – 'You have to let it go - the cancer - you can get it whether you smoke or not, you can get it from passive smoking. Not to say that it is just smoke, you can get it just from the air and anything else' - So I should let it go and not hit myself in the head with it." (Current smoker, DEN L17)

"I said, 'Have I caused this cancer through smoking?' So she started laughing. She [the doctor], said 'I'll explain, this type of cancer that you've got it's in women, more in women than in men.' She said, 'And it's not smoke related because people that don't smoke get this cancer more than people that do smoke so, no." (Current smoker, ENG L09)

While at least some of the smokers (and ex-smokers) in all countries alluded to feeling partly responsible for their LC, this was far more dominant in the English participants' accounts. English participants also described feeling the need to pre-empt and manage people's assumptions that the lung cancer must be smoking related. One man, who had not smoked for over four decades, explained that he had labelled his disease a 'non-smoking lung cancer' to distance himself from people's assumptions.

"I can be classed as a non-smoker, 45 years no smoking. So we found out that, you know, lung cancer does have a stigma to it in society. I went down, I just had been chatting to neighbours two doors down about what had happened and she said, "Oh, that will stop you

For peer review only - http://bmjopen.bmj.com/site/about/guidelines.xhtml

smoking, won't it." So we always describe it as "non-smoking lung cancer." (Ex-smoker, ENG L02)

Another described her pre-emptive tactic of explaining that she had stopped smoking a long time ago. *"Yeah. I think there is a little element [...], that you feel you know, you were stupid to smoke in the first place and you shouldn't have done. But then, look how many people smoke so [laughs]. Yeah, so, yeah, there is that little hint of, 'Well it's your own fault' sort of thing. So I'm very quick to say I haven't for 34 years."* (Ex-smoker, ENG L12)

Interestingly, while none of the Swedish or Danish participants said that they had stopped smoking shortly before consulting the GP, several of the English smokers said they gave up smoking before they consulted. The following participant became convinced that something was wrong when the breathlessness did not abate after stopping smoking: *"I gave up smoking. I had the, you know, something isn't right. I gave up smoking and then I got a chest infection. …. It was when I went back to the GP after the chest infection [um] because I still couldn't breathe. That underlying breathlessness was still there. It was then that [um] I just, something tells me that something's not right because I'm still feeling breathless and I packed in smoking."* (Recent ex-smoker, ENG L01)

DISCUSSION

Our study shows that, while there is high awareness of LC as a smoking related disease in all three countries, the 'smoking' frame is a more immediate and dominant part of the LC narrative in England and only in England did current smokers describe a reluctance to consult because of their smoking status. Our study draws attention to cross country differences in how cigarette smoking is framed in relation to discourses of responsibility for lung cancer. The

For peer review only - http://bmjopen.bmj.com/site/about/guidelines.xhtml

relationship between responsibility, blame and stigma is mediated by the health system and culture, even between seemingly comparable northern European countries.

Stigma, shame and blame matter, not only because they affect how the individual copes with their diagnosis, but also because they affect when and how people present their symptoms. There is no evidence to suggest that English participants were more aware of the relation between smoking and lung cancer than their Scandinavian counterparts, but only in the English accounts was this awareness offered as a reason for not consulting with symptoms. Earlier studies from England have shown that smoking contributes to delayed care seeking [2, 4, 15, 18, 28] and that smokers seek care late due to feelings of shame and personal responsibility [15, 18, 29]. Recent studies from England and Denmark also show that smokers are less likely to seek care for symptoms such as cough or hoarseness [30, 31]. Our comparative study suggests that the primacy given to smoking in the accounts mirrors cultural differences and norms about the connection between perceived blame and help seeking. The narratives suggest that in Sweden the smoking status of the individual is less relevant to the decision to seek care; indeed the 'Goldilocks Zone' for appropriate access to health care services may be broader in Sweden than in England [32].

Healthcare professionals who avoid victim blaming and treat the patient with kindness may influence people's attitudes to seeking care [17, 33]. As Corner argues [34] social processes are the means of culture and social organisation, shaping how people interpret and present symptoms to healthcare professionals, and affecting timely cancer diagnosis.

We also found more accounts of managing social interactions defensively, among participants from England, some of whom described acting defensively when others made assumptions about the cause of their lung cancer. This was seldom mentioned by the Danish and Swedish

For peer review only

participants. There are comparable findings from a Swedish study of patients diagnosed with another smoking-related disease chronical obstructive pulmonary diseases (COPD) [35].

Our study demonstrates that the sense of shame, blame and responsibility reported in earlier work on lung cancer experience in England and Scotland, is not an inevitable consequence of contracting a disease that is well known to be smoking related. There is nothing inevitable about whether and how this awareness influences the patients' decision to consult, nor affects the conduct of the consultation itself. Rather, it is rooted in particular relations between the public and publically funded healthcare.

Strengths and weakness of the study

This qualitative study with a cross-country comparison contributes to knowledge about the cultural context of health, care seeking and smoking among people recently diagnosed with lung cancer. A strength of this study is that the study design was informed by social science theories about stigma, shame and blame in the context of lung cancer. The interviews produced rich data from three Northern European countries with different lung cancer survival rates; the team approach enabled careful comparative analysis. Discussion of smoking was not prompted by the interviewers' questions, adding strength to our comparisons of when and how participants raised the topic.

Our study has limitations. While relatively large for a qualitative study we only interviewed participants in three countries. The results are based on patients' accounts and we did not observe patients' interactions with health care professionals or other people. However, as Greenhalgh [36] argues people's stories about illness are a critical window to its meaning systems and values and are nested with the narratives of society and culture.

For peer review only

CONCLUSION

Our study demonstrates that the sense of stigma, shame and blame reported in earlier work on lung cancer experience in England and Scotland, is not an inevitable consequence of contracting a disease that is related to an elective behaviour. There is nothing inevitable about whether and how this influences the patients' decision to consult, or affects the conduct of the consultation itself. Rather, the link between perceived responsibility and help seeking reflects particular relations between the public and publically funded healthcare. More responsive health systems would encourage a healthy lifestyle without deterring care seeking among those with concerning symptoms.

For peer review only

For peer review only - http://bmjopen.bmj.com/site/about/guidelines.xhtml

References:

1. Weiner B, Perry RP, and Magnusson J. An attributional analysis of reactions to stigmas. *J Pers Soc Psychol* 1988; 55(5):738-748.
2. Conlon A, Gilbert D, Jones B, et al. Stacked Stigma: Oncology Social Workers' Perceptions of the Lung Cancer Experience. *J Psychosoc Oncol* 2010;28(1):98-115.
3. Chapple A, Ziebland S, and McPherson A. Stigma, shame, and blame experienced by patients with lung cancer: qualitative study. *BMJ* 2004; 328(7454):1470.
4. Chambers S.K, Dunn J, Occhipinti S, et al. A systematic review of the impact of stigma and nihilism on lung cancer outcomes. *BMC Cancer* 2012;12(1):184.
5. Lebel S, and Devins GM. Stigma in cancer patients whose behavior may have contributed to their disease. *Future Oncol* 2008; 4(5):717-733.
6. Didkowska J, Wojciechowska U, Mańczuk M, et al. Lung cancer epidemiology: contemporary and future challenges worldwide. *Ann Transl Med* 2016;4(8):150.
7. WHO/Europe. *Tobacco Control Database for the WHO European Region*. 2016 [accessed 2018 5 Februari]; Available from: http://data.euro.who.int/tobacco/.
8. Walters S, Maringe C, Coleman M.P, et al. Lung cancer survival and stage at diagnosis in Australia, Canada, Denmark, Norway, Sweden and the UK: a population-based study, 2004-2007. *Thorax* 2013;68 (6):551-64.
9. Walters S, Benitez-Majano S, Muller P, et al. Is England closing the international gap in cancer survival? *Br J Cancer* 2015;113(5):848-860.
10. Butler J, Foot C, Bomb M, et al. The International Cancer Benchmarking Partnership: An international collaboration to inform cancer policy in Australia, Canada, Denmark, Norway, Sweden and the United Kingdom. *Health Policy* 2013;112(1–2):148-155.
11. Walter FM, Rubin G, Bankhead C, et al. Symptoms and other factors associated with time to diagnosis and stage of lung cancer: a prospective cohort study. *Br J Cancer* 2015;112 Suppl 1:S6-13.
12. Kleinman A. Patients and Healers in the Context of Culture: An Exploration of the Borderland between Anthropology, Medicine, and Psychiatry. Berkeey: University of California Press 1980;1-427.
13. Hajdarevic S, Hornsten Å, Sundbom E, et al. Patients' decision making in seeking care for suspected malignant melanoma. *J Nurs Healthc Chronic Illn* 2010;2(2):164-173.
14. Unger-Saldaña K and Infante-Castañeda CB. Breast cancer delay: A grounded model of help-seeking behaviour. *Soc Sci Med* 2011;72(7): 1096-1104.
15. Corner J, Hopkinson J, and Roffe L. Experience of health changes and reasons for delay in seeking care: A UK study of the months prior to the diagnosis of lung cancer. *Soc Sci Med*, 2006;62(6):1381-1391.
16. Carter-Harris L, Hermann CP, Schreiber j, et al. Lung Cancer Stigma Predicts Timing of Medical Help-Seeking Behavior. *Oncol Nurs Forum* 2014;41(3):E203-10.
17. Scott N, Crane M, Lafontaine M, et al. Stigma as a barrier to diagnosis of lung cancer: patient and general practitioner perspectives. *Prim Health Care Res Dev* 2015;16(6):618-622.
18. Tod AM, Craven J, Allmark P. Diagnostic delay in lung cancer: a qualitative study. *J Adv Nurs* 2008;61(3):336-343.
19. MacArtney J, Malmström M, Overgaard Nielsen T, et al. Patients' initial steps to cancer diagnosis in Denmark, England and Sweden: what can a qualitative, cross-country comparison of narrative interviews tell us about potentially modifiable factors? *BMJ Open* 2017;7(11).
20. Coyne IT. Sampling in qualitative research - Purposeful and theoretical sampling merging or clear boundaries. *J Adv Nurs* 1997;26(3):623-30.

For peer review only - http://bmjopen.bmj.com/site/about/guidelines.xhtml

21. Wyke S, Adamson J, Dixon D, et al. Consultation and illness behaviour in response to symptoms: a comparison of models from different disciplinary frameworks and suggestions for future research directions. *Soc Sci Med* 2013;86:79-87.
22. Levealahti H, Tishelman C, Ohlén J. Framing the onset of lung cancer biographically: narratives of continuity and disruption. *Psychooncology* 2007;16(5):466-73.
23. Andersen RS, Paarup B, Vedsted P et al. 'Containment' as an analytical framework for understanding patient delay: a qualitative study of cancer patients' symptom interpretation processes. *Soc Sci Med* 2010;71(2):378-85.
24. Evans J, Chapple A, Salisbury H, et al. It can't be very important because it comes and goes": patients' accounts of intermittent symptoms preceding a pancreatic cancer diagnosis: a qualitative study. *BMJ Open* 2014; 18;4(2):(e004215).
25. Ziebland S, Chapple A, Evans J. Barriers to shared decisions in the most serious of cancers: a qualitative study of patients with pancreatic cancer treated in the UK. *Health Expect* 2015;18(6):3302-12.
26. Chapple A, Ziebland S. Methodological and practical issues in cross-national qualitative research: Lessons from the literature and a comparative study of the experiences of people receiving a diagnosis of cancer. *Qual Health Res* 2018;28(5):789-799.
27. Pope C, Ziebland S, Mays N. Analysing qualitative data. *BMJ* 2000;320(7227):114-116.
28. Tod AM, Joanne R. Overcoming delay in the diagnosis of lung cancer: a qualitative study. *Nurs Stand* 2010;24(31):35-43.
29. Chatwin J, Sanders C. The influence of social factors on help-seeking for people with lung cancer. *Eur J Cancer Care* 2013;22(6):709-13.
30. Sele LM, Elnegaard S, Balasubramaniam K, et al. Lifestyle factors and contact to general practice with respiratory alarm symptoms—a population-based study. *BMC Fam Pract* 2016;17(1):47.
31. Friedemann Smith C, Whitaker KL, Winstanley K, et al. Smokers are less likely than non-smokers to seek help for a lung cancer 'alarm' symptom. *Thorax* 2016;71(7):659-661.
32. Ziebland S, Rasmussen BH, Hajdarevic S, et al. How wide is the Goldilocks Zone in your health system*? J Health Serv Res Policy* Forthcoming
33. Walton L, McNeill R, Stevens W, et al. Patient perceptions of barriers to the early diagnosis of lung cancer and advice for health service improvement*. Fam Practice* 2013; 30(4):436-444.
34. Corner J, Brindle L. The influence of social processes on the timing of cancer diagnosis: a research agenda*. J Epidemiol Community Health*, 2011;65(6):477-482.
35. Strang S, Farrell M, Larsson L-O, et al. Experience of guilt and strategies for coping with guilt in patients with severe COPD: a qualitative interview study. *J Palliat care* 2014;30(2):108-15.
36. Greenhalgh T. Cultural contexts of health: the use of narrative research in the health sector. Health Evidence Network synthesis report 49.WHO Regional Office for Europe: Copenhagen. 2016:1-67.

For peer review only - http://bmjopen.bmj.com/site/about/guidelines.xhtml

Other BMJ requirements (needed at various stages in the online submission process)

Acknowledgements

We are extremely grateful to the people who took part in this research, and to the study advisory panel, including patient and public representatives, who helped design the study and provided comments on an earlier draft of this manuscript. We also acknowledge the support of the National Institute for Health Research, through the Clinical Research Network, who helped recruit patients into the English arm of the study. We would also like to thank all those who helped to recruit participants: In Denmark we want to thank the Lung Cancer Patient organisation for assisting us in recruiting patients. In England we would like to thank the NHS Hospital Trusts that assisted with this study and Patients Active in Research: Thames Valley, along with the following charities who posted links or circulated our details on social media: Beating Bowel Cancer, Bowel Cancer UK, Roy Castle Lung Cancer Foundation, British Lung Foundation, and healthtalk.org. In Sweden we would like to thank the nurses and physicians who helped with recruitment.

Author Contributions

SZ developed the idea for the manuscript. SH wrote the first draft, with edits made by SZ, BHR, and TLOH. SZ, BR and TLOH contributed to the analysis meetings and provided comments on drafts.

Competing Interests

All authors have completed the ICMJE uniform disclosure form at www.icmje.org/coi_disclosure.pdf and declare: no support from any organisation for the submitted work; no financial relationships with any organisations that might have an interest

For peer review only

in the submitted work in the previous three years; no other relationships or activities that could appear to have influenced the submitted work.

Copyright Statement

The Corresponding Author has the right to grant on behalf of all authors and does grant on behalf of all authors, a worldwide licence to the Publishers and its licensees in perpetuity, in all forms, formats and media (whether known now or created in the future), to i) publish, reproduce, distribute, display and store the Contribution, ii) translate the Contribution into other languages, create adaptations, reprints, include within collections and create summaries, extracts and/or, abstracts of the Contribution, iii) create any other derivative work(s) based on the Contribution, iv) to exploit all subsidiary rights in the Contribution, v) the inclusion of electronic links from the Contribution to third party material where-ever it may be located; and, vi) licence any third party to do any or all of the above.

Ethics

This study was approved by ethical boards in each country separately in accordance to their requirements: in England by Research Ethics Service reference 14/NS/1035; Sweden by Regional Ethics Board, Lund, Sweden, reg. no 2014/819 and in Denmark The Biomedical Research Ethics Committee System Act does not apply to this project, as the project does not implicate the use of human biological materials. Standard ethical protocol according to the American Anthropological Association was followed.

Informed consent

All participants signed a consent form before taking part in an interview. Participants in England signed a second form after interview giving permission for their interview to be

For peer review only - http://bmjopen.bmj.com/site/about/guidelines.xhtml

available to researchers at the University of Oxford, and other universities under licence from Oxford, for use in research, teaching, and other publications.

Funding and independence of the researchers

This paper presents independent research funded by organisations from 3 European countries as follows:

In the UK, the study was supported by the National Awareness and Early Diagnosis Initiative (NAEDI), http://www.naedi.org.uk. The contributing partners include: Cancer Research UK; Department of Health, England; Economic and Social Research Council; Health and Social Care Research and Development Division, Pubic Health Agency, Northern Ireland; National Institute for Social Care and Health Research, Wales; and the Scottish Government. This funding also covered the costs associated with the comparative analysis meetings in Denmark and Sweden and funded translation of the Danish and Swedish material for publications.

In Denmark, the study was supported by the Research Centre for Cancer Diagnosis in Primary Care funded by The Danish Cancer Society and the Novo Nordic Foundation.

In Sweden, the study was supported by the Vårdal Foundation; the Strategic Research Program in Care Sciences (SFO-V), Umeå University; the Cancer Research Foundation in Northern Sweden; and from government funding of clinical research within the National Health Service, Sweden.

The views expressed in this paper are those of the authors, and not necessarily those of the NAEDI, Danish and Swedish funding partners.

For peer review only

Role of the sponsor

The study sponsor(s) or funder(s), had no role in the study design; the collection, analysis, and interpretation of data; writing of the report; or in the decision to submit the article for publication.

Access of researchers to all the data

All researchers in each country had access to all the data gathered in their own country. Interview transcripts were not translated and were not shared between countries, but Swedish and Danish interview extracts were translated by the bi-lingual researchers for the analytic discussions. The team lead in each country (SZ, RSA and BR) takes responsibility for the integrity of the data and the accuracy of the data analysis.

Transparency declaration

The lead author (the manuscript's guarantor) affirms that the manuscript is an honest, accurate, and transparent account of the study being reported; that no important aspects of the study have been omitted; and that any discrepancies from the study as planned (and, if relevant, registered) have been explained.

Data sharing statement

In Denmark the data are available for secondary analysis in conjunction with members of the research group named Comparative Cancer Experiences.

In England the participants gave informed consent for data to be copyrighted to the University of Oxford for secondary analysis, broadcasting, publication and teaching.

In Sweden the data are available for secondary analysis in conjunction with members of the research group named Comparative Cancer Experiences.

For peer review only

BMJ Open

A qualitative cross-country comparison of whether, when and how people diagnosed with lung cancer talk about cigarette smoking in narrative interviews

Journal:	*BMJ Open*
Manuscript ID	bmjopen-2018-023934.R1
Article Type:	Research
Date Submitted by the Author:	20-Aug-2018
Complete List of Authors:	Hajdarevic, Senada; Umeå University, Department of Nursing<br>Rasmussen, Birgit; The Institute for Palliative Care; Department of Health Sciences, Lund University<br>Overgaard Hasle, Trine; Research Centre for Cancer Diagnosis in Primary Care, Research Unit of General Practice, Aarhus University, Denmark<br>Ziebland, Sue; Health Experiences Research Group, Nuffield Department of Primary Care Health Sciences, University of Oxford
**Primary Subject Heading**:	Smoking and tobacco
Secondary Subject Heading:	Sociology, Qualitative research, General practice / Family practice, Health services research, Oncology
Keywords:	patients' experiences, lung cancer, smoking, qualitative comparative study, Organisation of health services < HEALTH SERVICES ADMINISTRATION & MANAGEMENT

SCHOLARONE™
Manuscripts

A qualitative cross-country comparison of whether, when and how people diagnosed with lung cancer talk about cigarette smoking in narrative interviews

Hajdarevic Senada[1], Senior Lecturer

Rasmussen Birgit H[2,3], Professor

Overgaard Hasle Trine L[4], Research assistant

*Ziebland Sue[5], Professor

[1]Department of Nursing, Umeå University, Umeå Sweden

[2] The Institute for Palliative Care, Lund University and Region Skåne, Sweden and Lund University, Skane University Hospital, Lund, Sweden

[3]Department of Clinical Sciences Lund, Surgery, Lund, Sweden

[4]Research Centre for Cancer Diagnosis in Primary Care, Research Unit of General Practice, Aarhus University, Aarhus Denmark

[5]Health Experiences Research Group, Nuffield Department of Primary Care Health Sciences, University of Oxford, UK.

Corresponding author:

Ziebland Sue,

Health Experiences Research Group, Nuffield Department of Primary Care Health Sciences, University of Oxford, UK.

sue.ziebland@phc.ox.ac.uk; phone: +44 (0)1865 289302

Keywords: health care, lung cancer, patients' experiences, qualitative study, smoking.

Word count, excluding title page, abstract, references, figures and tables: 2868 words

For peer review only - http://bmjopen.bmj.com/site/about/guidelines.xhtml

Abstract

Objectives: To compare and examine whether, when and how patients with lung cancer in three countries, with different survival rates, talk about cigarette smoking and its relationship with help seeking.

Design: A qualitative cross-country comparison with analysis of narrative interviews.

Setting: Participants in Sweden, Denmark and England were interviewed during 2015-2016. Interviews, using a narrative approach, were conducted in participants' home by trained and experienced qualitative researchers.

Participants: Seventy two men and women diagnosed with lung cancer were interviewed within six months of their diagnosis.

Results: The English participants, regardless of their own smoking status, typically raised the topic of smoking early in their interviews. Smoking was mentioned in relation to symptom appraisal and interactions with others, including health professionals. Participants in all three countries interpreted their symptoms in relation to their smoking status, but in Sweden (unlike England) there was no suggestion that this deterred them from seeking care. English participants, but not Swedish or Danish, recounted reluctance to consult healthcare professionals with their symptoms while they were still smoking, some gave up shortly before consulting. Some English patients described defensive strategies to challenge stigma or pre-empt other people's assumptions about their culpability for the disease. A quarter of the Danish and 40% of the Swedish participants did not raise the topic of smoking at any point in their interview.

For peer review only

Conclusion: The causal relationship between smoking and lung cancer is well-known in all three countries, yet this comparative analysis suggests that the links between a sense of responsibility, stigma and reluctance to consult is not inevitable. These findings help illuminate why English patients with lung cancer tend to be diagnosed at a later stage than their Swedish counterparts.

Article summary

Strengths of this study

The study design was informed by social science theories about stigma, shame and blame in the context of lung cancer.

Discussion of smoking was not prompted by the interviewers' questions, adding strength to our comparisons of whether, when and how participants raised the topic.

This analysis is based on an initially unanticipated point of comparison between the three countries, underlining the benefit of using an unstructured narrative approach to interviewing and attending to emergent themes during qualitative analysis

Limitations of this study

The results are based on patients' accounts and we did not observe patients' interactions with health care professionals or other people.

While relatively large for a qualitative study we only interviewed participants in three countries, all of which have publically funded health systems.

For peer review only

INTRODUCTION

Health problems which are known to be associated with certain elective ‘health behaviours’ can lead to stigma and victim-blaming [1]. A prominent example is lung cancer. Patients in England and Scotland have reported that, on hearing the diagnosis, other people assume that they are (or have been) heavy smokers [2-5]. Images of blackened lungs and television adverts including the personal testimonies of lung cancer patients voicing their regrets for smoking add to the impression of personal culpability [3]. A highly cited qualitative study published in the BMJ drew attention to the ‘stigma, shame and blame’ that many people with lung cancer experience due to the disease’s association with smoking [3]. This matters for several reasons, including because studies have shown that patients who fear being blamed are deterred from seeking help when they have symptoms [3, 4]. Yet, as we will show, there is no inevitability about the link between perceptions of responsibility, experience of stigma and a reluctance to consult. We draw on findings from our comparative qualitative study of experiences of lung cancer in Sweden, Denmark and England.

Lung cancer is the largest cancer related cause of death worldwide [6]. Denmark, UK and Sweden have similar population smoking rates (~18%, 20%, 20% respectively) [7]. International Cancer Benchmarking data have demonstrated that patients with lung cancer in Denmark and England are diagnosed at a more advanced stage than those in Sweden [8]. Sweden also continues to show a higher one-year survival for lung cancer than Denmark and England [9].

Possible explanations for why people in England and Denmark consult with later stage disease have been considered, including differences in how people interpret bodily sensations, access health care, describe their problem to their doctors and how the doctors respond. It has

For peer review only - http://bmjopen.bmj.com/site/about/guidelines.xhtml

also been demonstrated that English and Danish smokers with lung cancer symptoms consult their GPs later than non-smokers with the same symptoms [9-11].

Sociocultural aspects and characteristics of the health care system influence how and when people seek care [12]. Past experiences of contact with the health care services contribute to expectations and subsequent decisions to seek help [13-18]. Awareness of LC as a smoking related disease is high in all three countries. Smoking in restaurants, bars and workplaces has been banned in Sweden since 2005, Denmark since 2007 and England since 2007, with the legislation reinforcing the reduced social acceptance of smoking.

This paper draws on in-depth interviews collected during a cross- country qualitative comparative study of the lead up to a cancer diagnosis in three Northern European countries. The wider study sought to illuminate international variations in cancer stage at diagnosis by comparing people's accounts of their decisions and experiences of initially seeking care [19]. The current paper explores whether, when and how these interviewees with lung cancer talked about cigarette smoking during their open ended, narrative interviews. The topic of smoking was always raised by the participant, not the research team, enabling us to compare when and how they framed their talk about lung cancer and smoking.

METHOD

Participants and recruitment

We conducted a cross country comparative qualitative analysis of interviews collected within six months of a diagnosis of lung cancer (LC) in Denmark (n=22), England (n=20) and Sweden (n=30). Interviews were conducted during 2015-2016 as part of a wider qualitative

For peer review only - http://bmjopen.bmj.com/site/about/guidelines.xhtml

comparative study which also included interviews with patients with bowel cancer as well as lung cancer [19]. Purposive sampling [20] through hospital clinics (in Sweden) and also primary care, support organisations, via snowball-sampling and social media (in England and Denmark) sought comparable variation across gender, age, urban and rural locations, and pathway to diagnosis.

Data collection

In the wider comparative study participants gave informed consent and decided the place for their interview, usually their own home. The experienced qualitative researchers had backgrounds in nursing (Sweden), sociology (England) and anthropology (Denmark). A semi structured interview guide based on social science theories, and cancer research literature [3, 15, 21-25], was developed to enhance cross country comparisons [26]. The multi-disciplinary field work- team maintained close monthly contact by telephone conference during data collection. The whole team also met on several occasions for data analysis workshops which were held in all three countries. Further consideration of the challenges and solutions involved in cross-country qualitative work, drawing on examples from this project, have been reported [26]. All interviews began with an open-ended question: "*Could you start by telling me, in your own words and in as much detail as you want, about what has happened since you first started to suspect there might be a problem with your health?"* followed by flexibly used prompts from an interview guide. We deliberately avoided specific questions about whether the person smoked, or had ever smoked, cigarettes thus smoking status was always volunteered by the patient or marked as 'not known'. Interviews lasted between 45 and 90 minutes and were audio recorded for transcription and analysis.

For peer review only

Analysis

A specialist computer software (NVivo 10) was used to code, sort and retrieve data. The interview transcripts were read several times before coding. The cross country differences in talk about smoking arose as an unanticipated theme at one of our first analysis workshops when the PI (a co-author on the 2004 'Stigma, Shame and Blame' paper [3]) became aware that the centrality of smoking in British narratives about lung cancer, appeared to be less evident in the Swedish and Danish interviews. As a first step to test whether this impression was valid, we noted *whether* and *how early* in the interview the topic of smoking was first raised by the participant. Second, drawing on modified grounded theory and the technique of constant comparison we used thematic analysis [27] to consider *how* the participants talked about smoking during their interviews. People are described as 'current,' 'ex-smokers' or 'recent ex-smokers' on the basis of their narrative accounts. The researchers neither asked about smoking nor made assumptions about the smoking status – hence we describe smoking status as 'not known' if it was not raised by the participants.

Three authors (SH, SZ and BHR) used a data-driven coding framework developed through discussion at face to face workshop meetings and via phone conferences. We discussed and refined our interpretations of observed similarities and differences between the countries. For this paper direct quotes have been translated into English (and checked by BHR, the tri-lingual member of our team).

Patient involvement

Public and Patient Involvement (PPI) was conducted in accordance with good practice in each country. Lung cancer patients in England were involved in the whole process from the

For peer review only

preparation of funding application through the development of the study and discussion of preliminary results. PPI members in all three countries commented on the participant information sheets and the interview guide and advised on recruitment strategies.

RESULTS

The researchers' impression that there were differences between countries in the frequency, positioning and content of talk about smoking was confirmed when we compared the point in the interview at which participants raised the topic of smoking. Our thematic analysis of the talk about smoking draws on theories of responsibility [1] shame and blame [3] and is presented in relation to symptom appraisal, help seeking and communication with others.

Whether and how early smoking was raised by the participant

All but two of the English participants raised the topic of smoking, unprompted, in their interviews; this usually occurred within the opening sections or first 15% of the interview transcript. More than one quarter of the Danish participants and 40% of the Swedish participants did not raise the topic of smoking at all in their interviews; those who did were less likely than their English counterparts to raise it in the early part of their narrative (Table 1.).

Table1. Participant characteristics and cross-country comparison of *whether* and *how early* in the interview the topic of smoking was raised by the participant.

		Denmark	England	Sweden
Total number of interviewed participants		22	20	30
Women		8 (36%)	10 (50%)	15 (50%)
Age range (*year*)	31-50	0	2	2
	51-70	15	12	21
	71-90	7	6	7

For peer review only - http://bmjopen.bmj.com/site/about/guidelines.xhtml

Participants who raised the topic of smoking within the first 15% of the interview	9 (41%)	11(55%)	9 (30%)
Participants who raised the topic of smoking at any point in their interview	16 (73%)	18 (90%)	18 (60%)
Smoking NOT raised by participant during interview	6 (27%)	2 (10%)	12 (40%)

How participants talked about smoking

Participants raised the topic of smoking when talking about how they had interpreted their symptoms and when describing interactions with others, including how their health care professionals responded.

Smoking as a frame to interpret symptoms

Our first observation is that participants often raised the topic of smoking as if answering an (unasked) question, for example: *"But I mean, if I hadn't of broken me back they wouldn't have found (the cancer). And [er…pause]. The question is, 'Did you stop smoking?' well, Yeah."* (Ex- smoker, ENG ID18)

When describing decisions to consult, those who told us that they had never smoked or said they were longer term ex-smokers raised their (non)smoking status as part of their explanation for not expecting this diagnosis.

"I thought of course that it was pneumonia or something, so I never thought...as we all know, I have never smoked, I have never worked in smoky surroundings. I could never imagine that it could be something like that." (Never-smoker, SWE ID147)

For peer review only - http://bmjopen.bmj.com/site/about/guidelines.xhtml

Long-term ex-smokers often commented they had not been expecting lung cancer because they had stopped smoking many years ago. *"I mean, it never dawned on me that I was - lung cancer, didn't really figure. I have smoked when I was younger. That was 34 years ago."* (Ex-smoker, ENG ID12)

People who were still smokers, or who told us they had recently stopped, said they had interpreted and normalised their symptoms - a long-term cough was seen as a 'smoker's cough' and a degree of breathlessness as normal for a smoker.

'I had this persistent cough which I recognised as a smoker's cough, like [coughs] and it was constant'. (Ex-smoker, ENG ID05)

"You know that you smoke so it's normal with breathlessness when one was walking and exerted oneself a bit too much. So that was something you were expecting since you were smoking. So, therefore, you did not react in some specific way to it." (Current smoker, SWE ID105)

"And as long as I was smoking I was also coughing. But it was…it did not get worse. And it also disappeared when I quitted smoking, so I really did not have any indication." (Ex-smoker, SWE ID150)

Smokers also justified a reluctance to consult on the grounds that the sensations and symptoms they experienced were not sufficiently serious, that they felt too well, or the signs were not those they associated with lung cancer.

"I always thought lung cancer was due to smoking. [um] And I knew people who had lung cancer and they used to say to me, you know, [um] coughing up a lot of blood, things like that. I went, "Really." And I didn't have none of that. You see. So I never put it down to cancer." (Current smoker, ENG ID09)

For peer review only

"I know that I'm smoking and I've been smoking for many years, but I've always had good health and I've never coughed like many do in the morning for many years ... or ... I have never done that. So, I did not really think it could be cancer, because I had it great, and in principle I still have" (Current smoker, DK ID14)

Smoking as a frame when interacting with others

Accounts from some of the English smokers suggested that health care professionals who appeared to 'blame' smoking for the symptoms, had delayed taking action. *"... I said to the GP that my Dad and my Uncle had lung cancer and I thought they should have been a bit quicker to check it out. ... Basically it [the symptoms] was worse at night time and everything. First of all I think she [the GP] blamed it on my smoking."* (Current smoker, ENG ID03)

We also heard several accounts, in all three countries, of health care professionals who had worked hard to avoid any suggestion of blame or responsibility in relation to smoking.

"The doctor said – 'You have to let it go - the cancer - you can get it whether you smoke or not, you can get it from passive smoking. Not to say that it is just smoke, you can get it just from the air and anything else' - So I should let it go and not hit myself in the head with it." (Current smoker, DEN ID17)

"I said, 'Have I caused this cancer through smoking?' So she started laughing. She [the doctor], said 'I'll explain, this type of cancer that you've got it's in women, more in women than in men.' She said, 'And it's not smoke related because people that don't smoke get this cancer more than people that do smoke so, no." (Current smoker, ENG ID09)

For peer review only - http://bmjopen.bmj.com/site/about/guidelines.xhtml

In the following example a Swedish ex-smoker who voiced her concern that smoking might have been responsible was contradicted, kindly, though not entirely convincingly, by the doctor. *"Now we should not mix in smoking in this', the doctor said. And they were saying that in the order that I should not have such a guilty conscience, I can imagine. But she said that there were many non-smokers who get lung cancer,... but obviously they[doctors] think it[smoking] might have contributed, absolutely."* (Recent ex-smoker, SWE ID123)

While at least some of the smokers (and ex-smokers) in all countries alluded to feeling partly responsible for their lung cancer, this was far more dominant in the English participants' accounts. English participants also described feeling the need to pre-empt and manage people's assumptions that the lung cancer must be smoking related. One man, who had not smoked for over four decades, explained that he had labelled his disease a 'non-smoking lung cancer' to distance himself from people's assumptions.

"I can be classed as a non-smoker, 45 years no smoking. So we found out that, you know, lung cancer does have a stigma to it in society. I went down, I just had been chatting to neighbours two doors down about what had happened and she said, "Oh, that will stop you smoking, won't it." So we always describe it as "non-smoking lung cancer." (Ex-smoker, ENG ID02)

Another described her pre-emptive tactic of explaining that she had stopped smoking a long time ago. *"Yeah. I think there is a little element [...], that you feel you know, you were stupid to smoke in the first place and you shouldn't have done. But then, look how many people smoke so [laughs]. Yeah, so, yeah, there is that little hint of, 'Well it's your own fault' sort of thing. So I'm very quick to say I haven't for 34 years."* (Ex-smoker, ENG ID12)

For peer review only

Interestingly, while none of the Swedish or Danish participants said that they had stopped smoking shortly before consulting the GP, several of the English smokers said they gave up smoking before they consulted. The following participant became convinced that something was wrong when the breathlessness did not abate after stopping smoking: *"I gave up smoking. I had the, you know, something isn't right. I gave up smoking and then I got a chest infection. …. It was when I went back to the GP after the chest infection because I still couldn't breathe. That underlying breathlessness was still there. It was then that I just, something tells me that something's not right because I'm still feeling breathless and I packed in smoking."* (Recent ex-smoker, ENG ID01)

DISCUSSION

Our study shows that, while there is high awareness of lung cancer as a smoking related disease in all three countries, the 'smoking' frame is a more immediate and dominant part of the lung cancer narrative in England. In the English narratives, accounts of lung cancer are infused with references to smoking. Only in England did current smokers describe a reluctance to consult because of their smoking status or stopping smoking before they consulted the doctor. Our study draws attention to cross country differences in how cigarette smoking is framed in relation to discourses of responsibility [1] for lung cancer. The relationship between responsibility, blame and stigma reported by Chapple et al in 2004, appears to be mediated by the health system and culture, even between seemingly comparable northern European countries. Stigma, shame and blame matter, not only because they affect how the individual copes with their diagnosis, but also because they affect when and how people present their symptoms. There is no evidence to suggest that English participants were more aware of the relation between smoking and lung cancer than their Scandinavian

For peer review only

counterparts, but only in the English accounts was this awareness offered as a reason for not consulting with symptoms.

Earlier studies from England have shown that smoking contributes to delayed care seeking [2, 4, 15, 18, 28] and that smokers seek care late due to feelings of shame and personal responsibility [15, 18, 29]. Recent studies from England and Denmark also show that smokers are less likely than non-smokers to seek care for symptoms such as cough or hoarseness [30, 31]. Our comparative study suggests that the primacy given to smoking in the accounts mirrors cultural differences and norms about the connection between perceived blame and help seeking. The narratives suggest that in Sweden the smoking status of the individual is less relevant to the decision to seek care; indeed the ‘Goldilocks Zone’, within which patients feel it is appropriate to access to health care services [32], may be broader in Sweden than in England.

Healthcare professionals who avoid victim blaming and treat the patient with kindness may influence people’s attitudes to seeking care [17, 33]. As Corner argues [34] social processes are the means of culture and social organisation, shaping how people interpret and present symptoms to healthcare professionals, and affecting timely cancer diagnosis.

We also found more accounts of managing social interactions defensively, among participants from England, some of whom described acting defensively when others made assumptions about the cause of their lung cancer. This was seldom mentioned by the Danish and Swedish participants. There are comparable findings from a Swedish study of patients diagnosed with another smoking-related disease chronical obstructive pulmonary diseases (COPD) [35].

For peer review only - http://bmjopen.bmj.com/site/about/guidelines.xhtml

Strengths and weakness of the study

This qualitative study with a cross-country comparison contributes to knowledge about the cultural context of health, care seeking and smoking among people recently diagnosed with lung cancer. A strength of this study is that the study design was informed by social science theories about stigma, shame and blame in the context of lung cancer. The interviews produced rich data from three Northern European countries with different lung cancer survival rates; the team approach enabled careful comparative analysis. Discussion of smoking was not prompted by the interviewers' questions, adding strength to our comparisons of whether, when and how participants raised the topic. This analysis is based on an initially unanticipated point of comparison between the three countries, which would not have been evident had a more structured approach to the interviews been used, or had smoking status been amongst the standard questions. This underlines the benefit of using an unstructured narrative approach to interviewing and attending to emergent themes during qualitative analysis.

Our study has limitations. While relatively large for a qualitative study we only interviewed participants in three countries, all of which have publically funded health systems. The data were gathered as part of a larger study, the main results of which have been published in this journal [19]. The results are based on patients' accounts and we did not observe patients' interactions with health care professionals or other people. However, as Greenhalgh [36] argues people's stories about illness are a critical window to meaning systems and values and are nested with the narratives of society and culture.

CONCLUSION

By demonstrating differences between patients' narratives of lung cancer in England, Sweden and Denmark the study challenges the notion that there is any inevitability about the link

For peer review only - http://bmjopen.bmj.com/site/about/guidelines.xhtml

between responsibility, stigma and reluctance to seek care. There is nothing inevitable about whether and how this influences the patients' decision to consult, or how an individual's history of smoking cigarettes might affect the conduct of the consultation itself. Rather, the link between perceived responsibility and help seeking seems to reflect particular relations between the public and publically funded healthcare. The causal relationship between smoking and lung cancer is undeniable, yet the consequences of this awareness for patients seeking care is not uniform.

The study sheds light on findings from international comparisons of cancer survival by offering a new, potentially modifiable, explanation for why lung cancer patients in England may be diagnosed at a later stage.

For peer review only

References:

1. Weiner B, Perry RP, and Magnusson J. An attributional analysis of reactions to stigmas. *J Pers Soc Psychol* 1988; 55(5):738-748.

2. Conlon A, Gilbert D, Jones B, et al. Stacked Stigma: Oncology Social Workers' Perceptions of the Lung Cancer Experience. *J Psychosoc Oncol* 2010;28(1):98-115.

3. Chapple A, Ziebland S, and McPherson A. Stigma, shame, and blame experienced by patients with lung cancer: qualitative study. *BMJ* 2004; 328(7454):1470.

4. Chambers S.K, Dunn J, Occhipinti S, et al. A systematic review of the impact of stigma and nihilism on lung cancer outcomes. *BMC Cancer* 2012;12(1):184.

5. Lebel S, and Devins GM. Stigma in cancer patients whose behavior may have contributed to their disease. *Future Oncol* 2008; 4(5):717-733.

6. Didkowska J, Wojciechowska U, Mańczuk M, et al. Lung cancer epidemiology: contemporary and future challenges worldwide. *Ann Transl Med* 2016;4(8):150.

7. WHO/Europe. *Tobacco Control Database for the WHO European Region*. 2016 [accessed 2018 13th August]; Available from: http://www.euro.who.int/en/health-topics/disease- prevention/tobacco

8. Walters S, Maringe C, Coleman M.P, et al. Lung cancer survival and stage at diagnosis in Australia, Canada, Denmark, Norway, Sweden and the UK: a population-based study, 2004-2007. *Thorax* 2013;68 (6):551-64.

9. Walters S, Benitez-Majano S, Muller P, et al. Is England closing the international gap in cancer survival? *Br J Cancer* 2015;113(5):848-860.

10. Butler J, Foot C, Bomb M, et al. The International Cancer Benchmarking Partnership: An international collaboration to inform cancer policy in Australia, Canada, Denmark, Norway, Sweden and the United Kingdom. *Health Policy* 2013;112(1–2):148-155.

For peer review only - http://bmjopen.bmj.com/site/about/guidelines.xhtml

11. Walter FM, Rubin G, Bankhead C, et al. Symptoms and other factors associated with time to diagnosis and stage of lung cancer: a prospective cohort study. *Br J Cancer* 2015;112 Suppl 1:S6-13.

12. Kleinman A. Patients and Healers in the Context of Culture: An Exploration of the Borderland between Anthropology, Medicine, and Psychiatry. Berkeey: University of California Press 1980;1-427.

13. Hajdarevic S, Hornsten Å, Sundbom E, et al. Patients' decision making in seeking care for suspected malignant melanoma. *J Nurs Healthc Chronic Illn* 2010;2(2):164-173.

14. Unger-Saldaña K and Infante-Castañeda CB. Breast cancer delay: A grounded model of help-seeking behaviour. *Soc Sci Med* 2011;72(7): 1096-1104.

15. Corner J, Hopkinson J, and Roffe L. Experience of health changes and reasons for delay in seeking care: A UK study of the months prior to the diagnosis of lung cancer. *Soc Sci Med*, 2006;62(6):1381-1391.

16. Carter-Harris L, Hermann CP, Schreiber j, et al. Lung Cancer Stigma Predicts Timing of Medical Help-Seeking Behavior. *Oncol Nurs Forum* 2014;41(3):E203-10.

17. Scott N, Crane M, Lafontaine M, et al. Stigma as a barrier to diagnosis of lung cancer: patient and general practitioner perspectives. *Prim Health Care Res Dev* 2015;16(6):618-622.

18. Tod AM, Craven J, Allmark P. Diagnostic delay in lung cancer: a qualitative study. *J Adv Nurs* 2008;61(3):336-343.

19. MacArtney J, Malmström M, Overgaard Nielsen T, et al. Patients' initial steps to cancer diagnosis in Denmark, England and Sweden: what can a qualitative, cross-country comparison of narrative interviews tell us about potentially modifiable factors? *BMJ Open* 2017;7(11).

For peer review only

20. Coyne IT. Sampling in qualitative research - Purposeful and theoretical sampling merging or clear boundaries. *J Adv Nurs* 1997;26(3):623-30.

21. Wyke S, Adamson J, Dixon D, et al. Consultation and illness behaviour in response to symptoms: a comparison of models from different disciplinary frameworks and suggestions for future research directions. *Soc Sci Med* 2013;86:79-87.

22. Levealahti H, Tishelman C, Ohlén J. Framing the onset of lung cancer biographically: narratives of continuity and disruption. *Psychooncology* 2007;16(5):466-73.

23. Andersen RS, Paarup B, Vedsted P et al. 'Containment' as an analytical framework for understanding patient delay: a qualitative study of cancer patients' symptom interpretation processes. *Soc Sci Med* 2010;71(2):378-85.

24. Evans J, Chapple A, Salisbury H, et al. It can’t be very important because it comes and goes”: patients’ accounts of intermittent symptoms preceding a pancreatic cancer diagnosis: a qualitative study. *BMJ Open* 2014; 18;4(2):(e004215).

25. Ziebland S, Chapple A, Evans J. Barriers to shared decisions in the most serious of cancers: a qualitative study of patients with pancreatic cancer treated in the UK. *Health Expect* 2015;18(6):3302-12.

26. Chapple A, Ziebland S. Methodological and practical issues in cross-national qualitative research: Lessons from the literature and a comparative study of the experiences of people receiving a diagnosis of cancer. *Qual Health Res* 2018;28(5):789-799.

27. Pope C, Ziebland S, Mays N. Analysing qualitative data. *BMJ* 2000;320(7227):114-116.

28. Tod AM, Joanne R. Overcoming delay in the diagnosis of lung cancer: a qualitative study. *Nurs Stand* 2010;24(31):35-43.

For peer review only

29. Chatwin J, Sanders C. The influence of social factors on help-seeking for people with lung cancer. *Eur J Cancer Care* 2013;22(6):709-13.

30. Sele LM, Elnegaard S, Balasubramaniam K, et al. Lifestyle factors and contact to general practice with respiratory alarm symptoms—a population-based study. *BMC Fam Pract* 2016;17(1):47.

31. Friedemann Smith C, Whitaker KL, Winstanley K,, et al. Smokers are less likely than non-smokers to seek help for a lung cancer 'alarm' symptom. *Thorax* 2016;71(7):659-661.

32. Ziebland S, Rasmussen BH, Hajdarevic S, et al. How wide is the Goldilocks Zone in your health system? *J Health Serv Res Policy* Forthcoming

33. Walton L, McNeill R, Stevens W, et al. Patient perceptions of barriers to the early diagnosis of lung cancer and advice for health service improvement. *Fam Practice* 2013; 30(4):436-444.

34. Corner J, Brindle L. The influence of social processes on the timing of cancer diagnosis: a research agenda. *J Epidemiol Community Health*, 2011;65(6):477-482.

35. Strang S, Farrell M, Larsson L-O, et al. Experience of guilt and strategies for coping with guilt in patients with severe COPD: a qualitative interview study. *J Palliat care* 2014;30(2):108-15.

36. Greenhalgh T. Cultural contexts of health: the use of narrative research in the health sector. Health Evidence Network synthesis report 49.WHO Regional Office for Europe: Copenhagen. 2016:1-67.

For peer review only

Other BMJ requirements (needed at various stages in the online submission process)

Acknowledgements

We are extremely grateful to the people who took part in this research, and to the study advisory panel, including patient and public representatives, who helped design the study and provided comments on an earlier draft of this manuscript. We also acknowledge the support of the National Institute for Health Research, through the Clinical Research Network, who helped recruit patients into the English arm of the study. We would also like to thank all those who helped to recruit participants: In Denmark we want to thank the Lung Cancer Patient organisation for assisting us in recruiting patients. In England we would like to thank the NHS Hospital Trusts that assisted with this study and Patients Active in Research: Thames Valley, along with the following charities who posted links or circulated our details on social media: Beating Bowel Cancer, Bowel Cancer UK, Roy Castle Lung Cancer Foundation, British Lung Foundation, and healthtalk.org. In Sweden we would like to thank the nurses and physicians who helped with recruitment. Interviews in England were conducted by John MacArtney.

We would like to dedicate this paper to our colleague and co-applicant Alison Chapple PhD (1944-2018) whose work inspired this analysis. Alison was a lifelong non-smoker who was diagnosed with lung cancer in 2017, shortly after her retirement.

For peer review only

Author Contributions

SZ developed the idea for the manuscript. SH wrote the first draft, with edits made by SZ, BR, and TLOH. SH, SZ, BR and TLOH contributed to the analysis meetings and provided comments on drafts. SZ prepared the final draft and responded to the reviewer's comments.

Competing Interests

All authors have completed the ICMJE uniform disclosure form at www.icmje.org/coi_disclosure.pdf and declare: no support from any organisation for the submitted work; no financial relationships with any organisations that might have an interest in the submitted work in the previous three years; no other relationships or activities that could appear to have influenced the submitted work.

Copyright Statement

The Corresponding Author has the right to grant on behalf of all authors and does grant on behalf of all authors, a worldwide licence to the Publishers and its licensees in perpetuity, in all forms, formats and media (whether known now or created in the future), to i) publish, reproduce, distribute, display and store the Contribution, ii) translate the Contribution into other languages, create adaptations, reprints, include within collections and create summaries, extracts and/or, abstracts of the Contribution, iii) create any other derivative work(s) based on the Contribution, iv) to exploit all subsidiary rights in the Contribution, v) the inclusion of electronic links from the Contribution to third party material where-ever it may be located; and, vi) licence any third party to do any or all of the above.

Ethics

This study was approved by ethical boards in each country separately in accordance to their requirements: in England by Research Ethics Service reference 14/NS/1035; Sweden by Regional Ethics Board, Lund, Sweden, reg. no 2014/819 and in Denmark The Biomedical Research Ethics Committee System Act does not apply to this project, as the project does not implicate the use of human biological materials. Standard ethical protocol according to the American Anthropological Association was followed.

For peer review only

Informed consent

All participants signed a consent form before taking part in an interview. Participants in England signed a second form after interview giving permission for their interview to be available to researchers at the University of Oxford, and other universities under licence from Oxford, for use in research, teaching, and other publications.

Funding and independence of the researchers

This paper presents independent research funded by organisations from 3 European countries as follows:

In the UK, the study was supported by the National Awareness and Early Diagnosis Initiative (NAEDI), http://www.naedi.org.uk. The contributing partners include: Cancer Research UK; Department of Health, England; Economic and Social Research Council; Health and Social Care Research and Development Division, Pubic Health Agency, Northern Ireland; National Institute for Social Care and Health Research, Wales; and the Scottish Government. This funding also covered the costs associated with the comparative analysis meetings in Denmark and Sweden and funded translation of the Danish and Swedish material for publications.

In Denmark, the study was supported by the Research Centre for Cancer Diagnosis in Primary Care funded by The Danish Cancer Society and the Novo Nordic Foundation.

In Sweden, the study was supported by the Vårdal Foundation; the Strategic Research Program in Care Sciences (SFO-V), Umeå University; the Cancer Research Foundation in Northern Sweden; and from government funding of clinical research within the National Health Service, Sweden.

For peer review only

The views expressed in this paper are those of the authors, and not necessarily those of the NAEDI, Danish and Swedish funding partners.

Role of the sponsor

The study sponsor(s) or funder(s), had no role in the study design; the collection, analysis, and interpretation of data; writing of the report; or in the decision to submit the article for publication.

Access of researchers to all the data

All researchers in each country had access to all the data gathered in their own country. Interview transcripts were not translated and were not shared between countries, but Swedish and Danish interview extracts were translated by the bi-lingual researchers for the analytic discussions. The team lead in each country (SZ, RSA and BR) takes responsibility for the integrity of the data and the accuracy of the data analysis.

Transparency declaration

The lead author (the manuscript's guarantor) affirms that the manuscript is an honest, accurate, and transparent account of the study being reported; that no important aspects of the study have been omitted; and that any discrepancies from the study as planned (and, if relevant, registered) have been explained.

Data sharing statement

In Denmark the data are available for secondary analysis in conjunction with members of the research group named Comparative Cancer Experiences.

In England the participants gave informed consent for data to be copyrighted to the

For peer review only

University of Oxford for secondary analysis, broadcasting, publication and teaching.

In Sweden the data are available for secondary analysis in conjunction with members of the research group named Comparative Cancer Experiences.

For peer review only

Standards for Reporting Qualitative Research (SRQR)*

http://www.equator-network.org/reporting-guidelines/srqr/

Title and abstract

	Page/line no(s).
**Title** - Concise description of the nature and topic of the study Identifying the study as qualitative or indicating the approach (e.g., ethnography, grounded theory) or data collection methods (e.g., interview, focus group) is recommended	Page 1
**Abstract** - Summary of key elements of the study using the abstract format of the intended publication; typically includes background, purpose, methods, results, and conclusions	Page 2

Introduction

**Problem formulation** - Description and significance of the problem/phenomenon studied; review of relevant theory and empirical work; problem statement	P 6-7
**Purpose or research question** - Purpose of the study and specific objectives or questions	P7

Methods

**Qualitative approach and research paradigm** - Qualitative approach (e.g., ethnography, grounded theory, case study, phenomenology, narrative research) and guiding theory if appropriate; identifying the research paradigm (e.g., postpositivist, constructivist/ interpretivist) is also recommended; rationale**	P 8-9
**Researcher characteristics and reflexivity** - Researchers' characteristics that may influence the research, including personal attributes, qualifications/experience, relationship with participants, assumptions, and/or presuppositions; potential or actual interaction between researchers' characteristics and the research questions, approach, methods, results, and/or transferability	P8
**Context** - Setting/site and salient contextual factors; rationale**	P8
**Sampling strategy** - How and why research participants, documents, or events were selected; criteria for deciding when no further sampling was necessary (e.g., sampling saturation); rationale**	P8
**Ethical issues pertaining to human subjects** - Documentation of approval by an appropriate ethics review board and participant consent, or explanation for lack thereof; other confidentiality and data security issues	P26
**Data collection methods** - Types of data collected; details of data collection procedures including (as appropriate) start and stop dates of data collection and analysis, iterative process, triangulation of sources/methods, and modification of procedures in response to evolving study findings; rationale**	P8-9

For peer review only - http://bmjopen.bmj.com/site/about/guidelines.xhtml

**Data collection instruments and technologies** - Description of instruments (e.g., interview guides, questionnaires) and devices (e.g., audio recorders) used for data collection; if/how the instrument(s) changed over the course of the study	P 8-9
**Units of study** - Number and relevant characteristics of participants, documents, or events included in the study; level of participation (could be reported in results)	P8
**Data processing** - Methods for processing data prior to and during analysis, including transcription, data entry, data management and security, verification of data integrity, data coding, and anonymization/de-identification of excerpts	P8-9
**Data analysis** - Process by which inferences, themes, etc., were identified and developed, including the researchers involved in data analysis; usually references a specific paradigm or approach; rationale**	P8-9
**Techniques to enhance trustworthiness** - Techniques to enhance trustworthiness and credibility of data analysis (e.g., member checking, audit trail, triangulation); rationale**	P8-9

Results/findings

**Synthesis and interpretation** - Main findings (e.g., interpretations, inferences, and themes); might include development of a theory or model, or integration with prior research or theory	P10-15
**Links to empirical data** - Evidence (e.g., quotes, field notes, text excerpts, photographs) to substantiate analytic findings	P10-15

Discussion

**Integration with prior work, implications, transferability, and contribution(s) to the field -** Short summary of main findings; explanation of how findings and conclusions connect to, support, elaborate on, or challenge conclusions of earlier scholarship; discussion of scope of application/generalizability; identification of unique contribution(s) to scholarship in a discipline or field	P16-17
**Limitations** - Trustworthiness and limitations of findings	P17-18

Other

**Conflicts of interest** - Potential sources of influence or perceived influence on study conduct and conclusions; how these were managed	P25
**Funding** - Sources of funding and other support; role of funders in data collection, interpretation, and reporting	P28

*The authors created the SRQR by searching the literature to identify guidelines, reporting standards, and critical appraisal criteria for qualitative research; reviewing the reference lists of retrieved sources; and contacting experts to gain feedback. The SRQR aims to improve the transparency of all aspects of qualitative research by providing clear standards for reporting qualitative research.

For peer review only

**The rationale should briefly discuss the justification for choosing that theory, approach, method, or technique rather than other options available, the assumptions and limitations implicit in those choices, and how those choices influence study conclusions and transferability. As appropriate, the rationale for several items might be discussed together.

Reference:

O'Brien BC, Harris IB, Beckman TJ, Reed DA, Cook DA. **Standards for reporting qualitative research: a synthesis of recommendations.** *Academic Medicine*, Vol. 89, No. 9 / Sept 2014 DOI: 10.1097/ACM.0000000000000388

For peer review only
